# Atrophic and Metaplastic Progression in the Background Mucosa of Patients with Gastric Adenoma

**DOI:** 10.1371/journal.pone.0169456

**Published:** 2017-01-10

**Authors:** Hee Kyong Na, Charles J. Cho, Suh Eun Bae, Jeong Hoon Lee, Young Soo Park, Ji Yong Ahn, Do Hoon Kim, Kee Don Choi, Ho June Song, Gin Hyug Lee, Se Jin Jang, Hwoon-Yong Jung

**Affiliations:** 1 Department of Internal Medicine, University of Ulsan College of Medicine, Asan Medical Center, Seoul, South Korea; 2 Department of Biomedical Science, University of Ulsan College of Medicine, Asan Medical Center, Seoul, South Korea; 3 Health Screening and Promotion Center, University of Ulsan College of Medicine, Asan Medical Center, Seoul, South Korea; 4 Department of Pathology, University of Ulsan College of Medicine, Asan Medical Center, Seoul, South Korea; Gentofte Hospital, DENMARK

## Abstract

**Background:**

In patients with adenoma, assessing premalignant changes in the surrounding mucosa is important for surveillance. This study evaluated atrophic and metaplastic progression in the background mucosa of adenoma or early gastric cancer (EGC) cases.

**Methods:**

Among 146 consecutive patients who underwent endoscopic resection for intestinal-type gastric neoplasia, the adenoma group included 56 patients with low-grade dysplasia and the ECG group included 90 patients with high-grade dysplasia or invasive carcinoma. For histology, 3 paired biopsies were obtained from the antrum, corpus lesser curvature (CLC), and corpus greater curvature (CGC). Serological atrophy was determined based on pepsinogen A (PGA), progastricsin (PGC), gastrin-17, and total ghrelin levels. Topographic progression of atrophy and/or metaplasia was staged using the operative link on gastritis assessment (OLGA) and operative link on gastric intestinal metaplasia assessment (OLGIM) systems.

**Results:**

Rates of moderate-to-marked histological atrophy/metaplasia in patients with adenoma were 52.7%/78.2% at the antrum (*vs*. 58.8%/76.4% in EGC group), 63.5%/75.0% at the CLC (*vs*. 60.2%/69.7% in EGC group), and 10.9%/17.9% at the CGC (*vs*. 5.6%/7.8% in EGC group). Serological atrophy indicated by PGA and PGC occurred in 23.2% and 15.6% of cases in the adenoma and ECG groups, respectively (*p* = 0.25). Mean serum gastrin-17 concentrations of the adenoma group and EGC group were 10.4 and 9.0 pmol/L, respectively (*p* = 0.54). Mean serum total ghrelin levels were 216.6 and 209.5 pg/mL, respectively (*p* = 0.71). Additionally, between group rates of stage III–IV OLGA and OLGIM were similar (25.9% *vs*. 25.0%, *p* = 0.90; 41.8% *vs*. 44.9%, *p* = 0.71, respectively).

**Conclusions:**

Atrophic and metaplastic progression is extensive and severe in gastric adenoma patients. A surveillance strategy for metachronous tumors should be applied similarly for patients with adenoma or EGC.

## Introduction

Gastric mucosal atrophy and intestinal metaplasia are associated with an increased risk of gastric cancer because they represent the background in which adenomas and intestinal-type gastric adenocarcinomas develop [1]. In a model of gastric carcinogenesis proposed by Correa et al., the intestinal type of gastric cancer results from progressive changes in the gastric mucosa, beginning with chronic gastritis and followed by atrophic gastritis and intestinal metaplasia [2,3]. Earlier studies have established that the relative risk of gastric cancer was 4.9 in severe atrophic gastritis when compared with none or mild atrophic gastritis, and was 6.4 in metaplastic gastritis compared with stomachs that lacked intestinal metaplasia [4,5].

Gastric adenoma is considered to be a premalignant lesion, even though an “adenoma-carcinoma sequence” in the stomach has not yet been established. Recent guideline recommends that all gastric adenoma should be removed when safe to do [6] because it can transform into invasive carcinoma and sometimes may already contain internal malignant cells [7–9]. However, the progression of atrophy and intestinal metaplasia in the background mucosa of patients with gastric adenoma has not yet been fully characterized nor has a surveillance strategy after endoscopic resection (ER) has been established. Herein, we investigated the extent and severity of atrophy and metaplasia in patients with gastric adenoma by comparing them to those patients with EGC.

## Patients and Methods

### Study Patients

Data were retrieved from the medical records of 165 consecutive patients with intestinal-type early gastric neoplasms who underwent endoscopic resection (ER) between December 2006 and September 2007 at Asan Medical Center Seoul, South Korea. The recommended indications for ER were as follows: 1) gastric adenoma with risk factors for malignant transformation, such as a depressed morphology, surface erythema, or a size ≥ 1 cm; 2) an intraepithelial tumor with high grade dysplasia of any size; and 3) a differentiated adenocarcinoma ≤ 3 cm in size that is mucosa-confined without an ulcer. Tumor location was divided into the upper, middle, and lower third by the lines connecting the trisected points the lesser and greater curvatures [10]. A total of 19 patients were excluded from the analysis for the following reasons: 7 for severe concomitant illness, 5 for previous *Helicobacter pylori* eradication therapy, 3 for previous intra-abdominal surgery, and 4 for taking acid-suppressive drugs in the previous 14 days. Ultimately, a total of 146 patients were included in our analysis. Patients with adenocarcinoma were also evaluated for either regional lymph node or distant metastasis using abdominal computed tomography. The study protocol was approved by the Institutional Review Board of Asan Medical Center, Seoul, Korea, and written informed consent was obtained from all participants.

### Histological Analysis of Chronic Atrophic Gastritis and Metaplasia

A total of 3 separate pairs of biopsies were obtained using elongated large-cup forceps (Olympus FB-24k-1; Olympus, Tokyo, Japan) for histological examinations of chronic atrophic gastritis and intestinal metaplasia. These biopsies included two from the antrum, two from the upper corpus lesser curvature (CLC), and two from the upper corpus greater curvature (CGC). Biopsy specimens were stained with hematoxylin and eosin (H&E) and Wright-Giemsa, and stained sections were examined by two experienced gastrointestinal pathologists (Y.S. Park and S.J. Jang) who had no knowledge of the original diagnosis. Histological parameters were assessed based on the updated Sydney system by grading with a 4-point visual analogue scale that ranged from 0 (absent) to 3 (marked) for each specimen [11]. The topographic pattern of chronic inflammation was classified as antrum-dominant gastritis, pan-gastritis, or corpus-dominant gastritis. The gastritis stage was also assessed using the operative link on gastritis assessment (OLGA) and operative link on gastric intestinal metaplasia assessment (OLGIM) staging systems [12,13]. The antrum score was calculated from the antrum, which was considered to be the non-oxyntic gastric mucosa, and the corpus score was calculated based on the CGC, which was defined as the oxyntic gastric mucosa. OLGA and OLGIM staging scores were calculated using a combination of both the antrum and corpus score.

### Measurements of Serum Pepsinogen A, Progastricsin, Gastrin-17, and Total Ghrelin

Serum samples were collected after overnight fasting at 8 AM on the day of ER and then were stored at –70°C until samples were used. Serum concentrations of pepsinogen A (PGA), progastricsin (pepsinogen C, PGC), and amidated gastrin-17 concentrations were measured using specific enzyme immunoassay kits (Pepsinogen A, Progastricsin, and Gastrin-17 EIA Test Kits; Biohit Plc., Helsinki, Finland). Serologic atrophy was defined as PGA <25 μg/L and/or or a PGA / PGC ratio < 3 [14,15]. Serum levels of total ghrelin were measured by ELISA (Human Ghrelin ELISA kit, total; EMD Millipore Corp., Billerica, MA).

### Diagnosis of *H*. *pylori* Infection

Histological diagnoses of *H*. *pylori* infection were based on Wright—Giemsa staining for each pair of biopsy specimens from the antrum, upper CLC, and upper CGC. Biopsy specimens were collected during the initial inspection of the gastric mucosa prior to the ER procedure. Additional biopsy specimens for rapid urease testing using a urease reaction kit (Hp Kit; Chongkundang Pharm. Corp., Seoul, Korea) were obtained from the antrum and upper CGC. At least two positively scored specimens on histology and/or urease tests at any biopsy site were required for the positive diagnosis of *H*. *pylori* infection.

### Statistical Analysis

Baseline variables are presented as numbers (with percentages) and means (with the standard deviation [SD]). Continuous variables were compared using Student’s *t*-test and categorical variables were compared using the *χ*^2^ test or Fisher’s exact test. Statistical analyses were performed using the SPSS software package, version 21 (SPSS Inc., Chicago, IL). All statistical calculations were two-sided, and a *p*-value <0.05 was used as a threshold for statistically significant differences.

## Results

### Patients and Tumor Characteristics

Among the 146 study patients, 56 (38.4%) were in the adenoma group and 90 (61.6%) were in the EGC group based on the final pathological analysis of the resected specimen. Among the 90 patients in the EGC group, 26 patients exhibited high-grade dysplasia (HGD), while 64 patients had invasive carcinoma. There were 21 patients (27.2%) with an adenoma at the time of initial biopsy that was found to have high-grade dysplasia or invasive cancer. A comparison of the clinico-pathological characteristics of the adenoma and EGC groups is presented in Table 1. *H*. *pylori* positivity did not differ between adenoma (62.5%) and EGC (61.1%) patients (*p* = 0.867). Rates of serological atrophy, as determined by PGA < 25 μg/L and/or PGA / PGC < 3, were estimated to be 23.2% and 15.6%, respectively (*p* = 0.246). The proportion of patients with synchronous lesions at the time of the procedure was similar between the adenoma and EGC groups (16.1% *vs*. 18.9%, *p* = 0.665).

**Table 1:** Comparison of Clinicopathological Features in the Adenoma and EGC Groups.

	Total	Adenoma group	EGC group	*P*-value
**Number**	146	56 (38.4)	90 (61.6)	
**Age, year (mean ± SD)**	63.0 ± 8.6	61.7 ± 8.0	63.8 ± 8.9	0.150
**Gender, male (%)**	104 (71.2)	36 (64.3)	68 (75.6)	0.144
**Family history of gastric cancer (%)**	27 (18.6)	9 (16.1)	18 (20.2)	0.532
**Smoking (%)**	85 (58.2)	31 (55.4)	54 (60.0)	0.580
**Alcohol consumption (%)**	50 (34.2)	15 (26.8)	35 (38.9)	0.134
***H*. *pylori* infection (%)**	90 (61.6)	35 (62.5)	55 (61.1)	0.867
**Tumor size, cm (mean ± SD)**	1.7 ± 1.2	1.6 ± 1.4	1.8 ± 1.1	0.287
**Tumor location**				0.861
**Upper third**	7 (4.8)	2 (3.6)	5 (5.6)	
**Middle third**	23 (15.8)	9 (16.1)	14 (15.6)	
**Lower third**	116 (79.5)	45 (80.4)	71 (78.9)	
**Multiplicity**	26 (17.8)	9 (16.1)	17 (18.9)	0.665
**Serologic atrophy**^a^ **(%)**	27 (18.5)	13 (23.2)	14 (15.6)	0.246

EGC, early gastric cancer.

Continuous variables are presented as means ± SD.

^a^ Serological atrophy was defined as pepsinogen A < 25 μg/L and/or a pepsinogen A / progastricsin ratio < 3.

### Histologic Evaluations of Atrophy and Metaplasia

**Fig 1:**
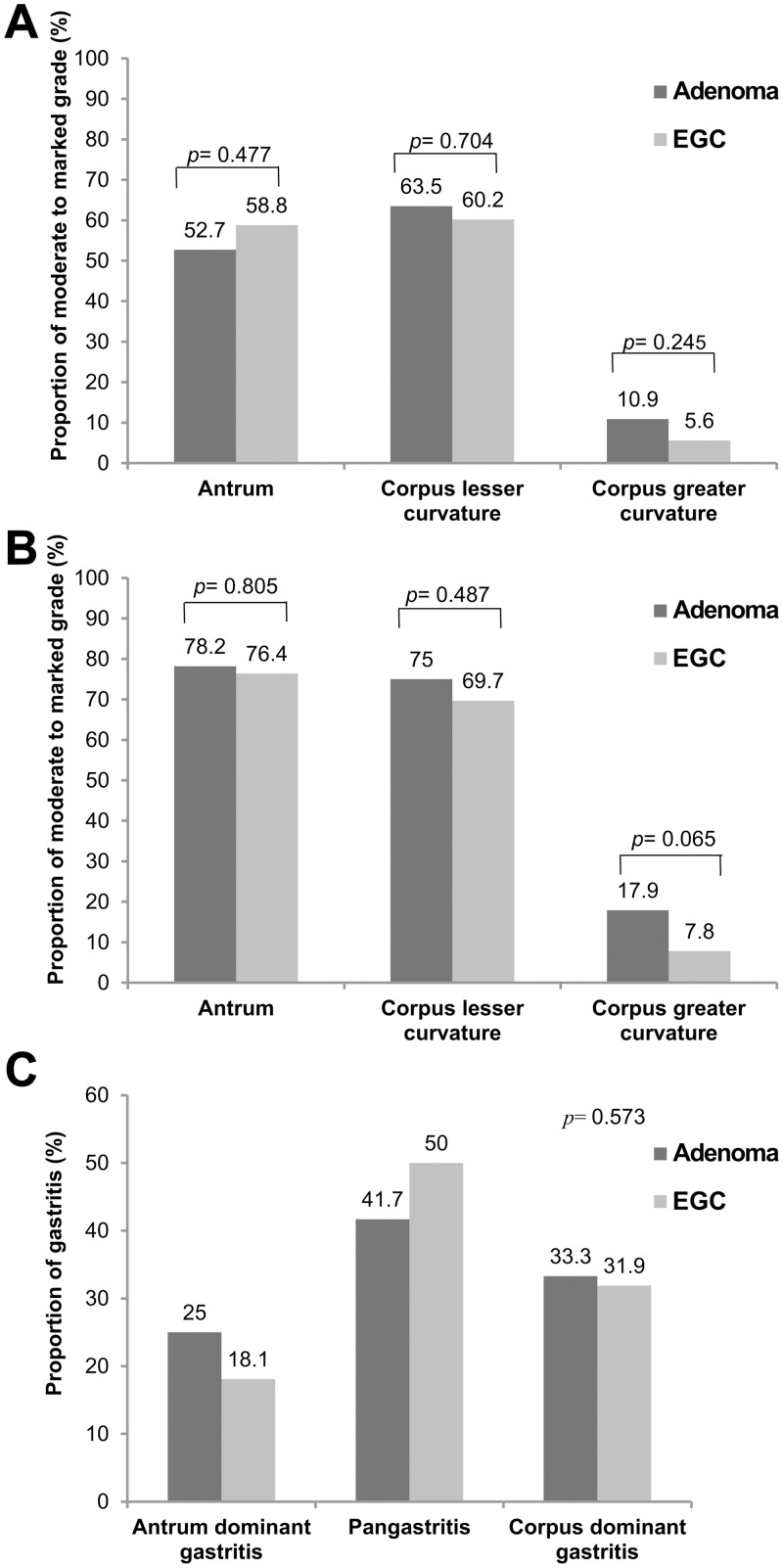
Histological comparisons of gastric adenoma and the early gastric cancer groups. (A-B) Proportion of lesions of a moderate to marked grade for atrophy and metaplasia according to the biopsy sites. (C) Pattern of gastritis.

The proportion of graded moderate-to-marked atrophy of the antrum (52.7% *vs*. 58.8%, *p* = 0.477), CLC (63.5% *vs*. 60.2%, *p* = 0.704), and CGC (10.9% *vs*. 5.6%, *p* = 0.245) did not differ between the adenoma and EGC groups (Fig 1A). Additionally, the frequencies of graded moderate-to-marked metaplasia of the antrum (78.2% *vs*. 76.4%, *p* = 0.805), CLC (75.0% vs. 69.7%, *p* = 0.487), and CGC (17.9% *vs*. 7.8%, *p* = 0.065) were also similar between the adenoma and EGC groups (Fig 1B). The topographic pattern of chronic inflammation is shown in Fig 1C. Regarding the distribution of gastritis patterns, there was no significant difference between the adenoma and EGC groups (*p* = 0.573) for antrum-dominant gastritis (25.0% vs. 18.1%), pangastritis (41.7% *vs*. 50.0%), or corpus-dominant gastritis (33.3% *vs*. 31.9%).

### Serological Atrophy Based on Pepsinogen A, Progastricsin, Gastrin-17, and Total Ghrelin

**Fig 2:**
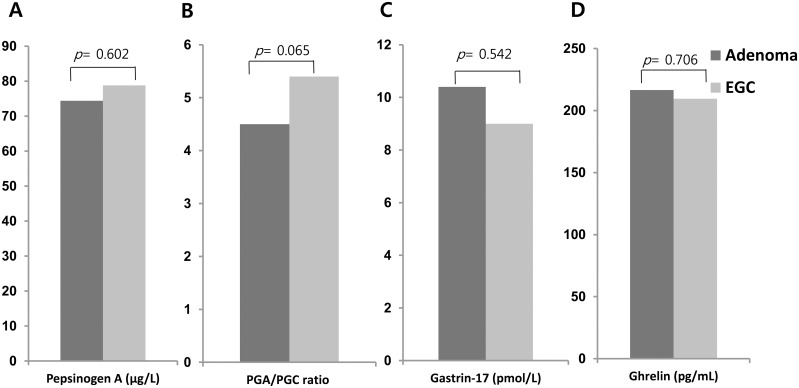
Serological comparisons of gastric adenoma and the early gastric cancer groups. (A) Pepsinogen A. (B) Pepsinogen A/Progastricsin ratio. (C) Gastrin-17. (D) Total ghrelin levels.

Fig 2 shows the mean levels of serological atrophy between the two groups. There was no difference in the serum concentrations of PGA or PGA/PGC ratio between the adenoma (PGA, 74.4±53.5 μg/L and PGA/PGC ratio, 4.5±2.7) and EGC groups (PGA, 78.8±47.1 μg/L and PGA/PGC ratio, 5.4±3.2; *p* = 0.602 and p = 0.065, respectively). Additionally, the mean serum gastrin-17 level failed to show significant difference between the two groups (10.4±13.4 pmol/L *vs*. 9.0±12.6 pmol/L, respectively; *p* = 0.542). The serum concentration of total ghrelin also did not differ between the two groups (216.6±124.2 pg/mL *vs*. 209.5±100.7 pg/mL; *p* = 0.706).

### Distribution of the OLGA and OLGIM Stages

**Fig 3:**
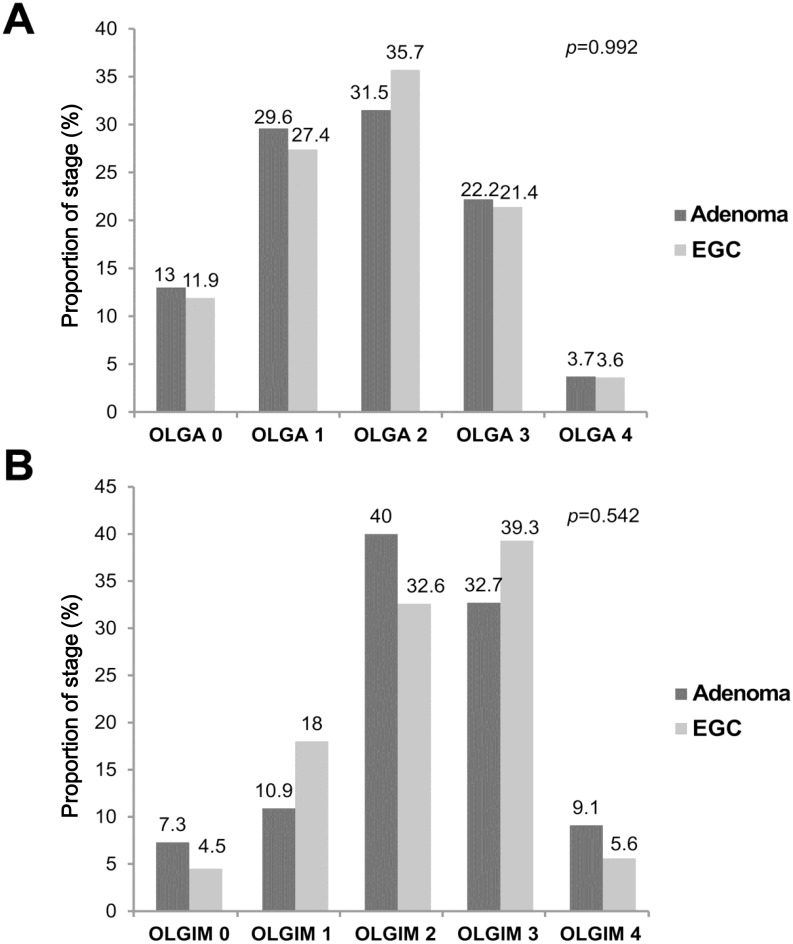
Frequency of lesions by gastritis stage. (A) Operative link on gastritis assessment (OLGA). (B) Operative link on gastric intestinal metaplasia (OLGIM).

The distribution of the OLGA and OLGIM stages is shown in Fig 3. No significant difference was detected between the frequencies of the OLGA (*p* = 0.992) and OLGIM (*p* = 0.542) stages between the two groups. Furthermore, between the adenoma and EGC groups, the proportions of cases of stage III–IV OLGA (25.9% *vs*. 25.0%, *p* = 0.903) and OLGIM (41.8% *vs*. 44.9%, *p* = 0.713) disease were similar.

### Development of Metachronous Tumors during Follow-up

Among the 146 patients, 40 patients failed regular follow-up: 12 patients who underwent subsequent gastrectomy due to non-curative resection and 28 patients who referred to local hospital with surveillance duration of less than 12 months. Then, among the 106 patients, 7 metachronous tumors occurred in 7 patients (6.6%). Specifically, 2 metachronous tumors (2 adenomas) occurred in 2 patients of 46 gastric adenoma patients (4.3%), and 5 metachronous tumors (4 EGCs and 1 adenoma) occurred in 5 of 60 EGC patients (8.3%; *p* = 0.696). Mean follow-up period of the adenoma group (60.7 ± 34.3 months) and EGC group (60.0 ± 30.2 months) did not differ. (*p* = 0.920).

## Discussions

In the East countries, especially in Korea and Japan, prevalence of *H*. *pylori* infection and incidence of gastric cancer are high. According the data of the Cancer Registry at the Korean National Cancer Center, the age-standardized incidence of gastric cancer during 1999–2013 period in South Korea was 55.3 per 100,000 person-years for men and 22.4 for women [16]. The prevalence of *H*. *pylori* infection in Korean screening population has been report as still high as 50–65% in the recent studies [17–20]. In addition, atrophic gastritis and intestinal metaplasia are reported to be found in 40.7% and 12.5% of the patients undergoing screening endoscopy [21]. In a Korean age- and sex-matched case-control study evaluating OLGA and OLGIM staging system, 26.6% of healthy control showed OLGA stage III-IV and 14.5% of the control group showed OLGIM stage III-IV [20]. These results suggest that the general population who has a high risk of developing gastric cancer also show high prevalence of pre-malignant gastric mucosal changes.

Our present study findings revealed that patients with gastric adenoma exhibited as much extensive and severe progression of atrophy and intestinal metaplasia as patients with EGC did. Premalignant changes in the background mucosa of these patients appeared to continue up to the end-stage of chronic gastritis. Because *H*. *pylori*-induced gastritis is typically acquired in childhood, the gastric mucosal damage caused by this bacterium accumulates over a long period of time and background mucosal changes are often advanced by the time adenoma or EGC occurs. A strength of our present study was that the comparisons of the surrounding mucosa were performed using various methods, and our analysis showed consistent results.

The findings of a previous retrospective study that analyzed the incidence of metachronous gastric cancer with 947 patients (361 patients with adenoma, 586 patients with EGC) who underwent ER are consistent with the results of our present study [22]. In that earlier study, the incidence of gastric cancer after ER did not differ between the adenoma (14.4 cases per 1000 person-years) and EGC groups (18.4 cases per 1000 person-years, *p* = 0.309) over a median follow-up of 28 months. Additionally, the degree of endoscopic atrophy and initial *H*. *pylori* infection rate did not significantly differ between the two groups. Therefore, the authors suggested that less-invasive forms of gastric neoplasia, such as adenoma, might not have a lower carcinogenic background when compared with more-invasive forms, such as EGC. In another study that investigated the histologic features of the surrounding mucosa in 118 patients with gastric adenoma, 60 patients with *H*. *pylori*-associated gastric adenoma were matched with the same number of patients with *H*. *pylori*-associated intestinal type EGC [23]. The degree and activity of *H*. *pylori* gastritis and intestinal metaplasia was similar in both groups. The authors of that report stated that when *H*. *pylori* was detected, gastritis was severe in the antrum and corpus. Additionally, intestinal metaplasia was a common finding in both adenoma and EGC patients.

In *H*. *pylori* gastritis, atrophic changes appear earlier at the level of the anglular (transitional mucosa), and then later involve the mucus-secreting antral compartment. Subsequently, atrophic changes extend proximally from the antrum to corpus, leading to the clinicopathological features of diffuse atrophic pan-gastritis [24]. In such cases, the percentages of *H*. *pylori*-infection are often low. Because of the loss of acid production, *H*. *pylori* cannot grow and may not be detectable, even though they had previously initiated the mucosal damage. In our present study, the percentage of patients with positive *H*. *pylori* infection was similar (~60%) between the two groups, which might allude to the similar extent of atrophic gastritis in the two groups.

In our present analysis, we used serological markers to compare atrophic changes between the two study groups. Serum PGA and PGC profiles are well established non-invasive biomarkers for gastric atrophy. The utility of serum PGA and PGC tests for the detection of subjects who are at high risk of gastric cancer with atrophic gastritis has been documented in many countries [25–27]. In our present study, serologic atrophy was identified in 18.5% of patients, and the rate was similar in both groups. Gastrin-17, another noninvasive serologic marker, has been shown to detect antral atrophy [28–30]. However, the performance of gastrin-17 test alone is not enough to detect atrophy and the test is mostly used with combination of a test-pannel including PGA and PGC [30,31]. Total ghrelin, a recently reported serologic marker, has been previously shown to be related to histological atrophy [32–35]. In an earlier study of 220 subjects, including patients both with and without atrophic gastritis, plasma ghrelin concentrations decreased along with the extent of atrophic changes in the gastric mucosa, irrespective of *H*. *pylori* infection [32]. In another study that assessed correlations between ghrelin levels and the histologic severity and topographical extent of chronic gastritis, ghrelin levels progressively decreased from a normal state, to antrum-predominant gastritis, to pan-gastritis, and finally to corpus-dominant gastritis [33]. In our previous study, we reported that total ghrelin levels were closely correlated with both PGA concentrations and the PGA/PGC ratio [35]. Our present findings revealed no significant difference in the serum PGA/ PGC ratio, gastrin-17 or ghrelin levels between the adenoma and EGC groups, which also supports the findings of our histological analysis.

The association of an advanced OLGA or OLGIM stage (stage III and IV) with an increased risk of gastric cancer has been established in multiple studies [20,36,37]. In a retrospective cross-sectional study that was performed with 474 gastric cancer patients along with age-and sex-matched healthy control patients, both OLGA and OLGIM stages III–IV were more prevalent among gastric cancer patients (26.6% vs. 46.2%, *p* < 0.001) and also were significantly associated with an increased risk of gastric cancer (odds ratio, 2.09; *p* = 0.008 and 2.04; *p* = 0.014, respectively) [20]. Similar proportions of advanced OLGA and OLGIM stages in the adenoma and EGC groups of our present study cohort may indicate that the risk of developing subsequent gastric cancer is similarly high in patients with adenoma and EGC.

In our present series, many (27.2%) lesions initially diagnosed as adenoma were ultimately diagnosed as HGD or EGC. Previous studies of the discrepancy between the results of forceps biopsy and ER found that 24.0–34.0% of gastric adenoma cases were ultimately diagnosed as HGD or EGC by the final pathological assessment [38–41]. A forceps biopsy may obtain insufficient tissue, so analyses of gastric adenoma tissue specimens cannot exclude the presence of invasive carcinoma. A large tumor size, surface erythema, and a depressed morphology are known to be predictive factors that are suggestive of malignant transformation of gastric adenoma [39,40,42,43]. In the report that analyzed 236 gastric adenoma lesions, a depressed morphology, surface erythema, and a size ≥1 cm were each significantly associated with HGD or carcinoma [39]. Moreover, the sensitivities of a negative predictive value for ≥1 risk factors were 93.8% and 90.9% for HGD and carcinoma, respectively. Therefore, ER should be performed for cases of gastric adenoma with such endoscopic features. Furthermore, an endoscopic surveillance strategy for patients with gastric adenoma after ER should be applied equally to all patients with EGC.

In conclusion, the extent and severity of atrophic and metaplastic changes in the background mucosa do not differ between those patients with adenoma and EGC. To detect metachronous tumors after ER, endoscopic surveillance of patients with gastric adenoma should be applied similarly to those patients with EGC.

## Supporting Information

S1 File Raw data of the study population. (XLS)
